# Translational Attenuation Mechanism of *ErmB* Induction by Erythromycin Is Dependent on Two Leader Peptides

**DOI:** 10.3389/fmicb.2021.690744

**Published:** 2021-06-28

**Authors:** Shasha Wang, Kai Jiang, Xinyue Du, Yanli Lu, Lijun Liao, Zhiying He, Weizhi He

**Affiliations:** ^1^Institute for Regenerative Medicine, Shanghai East Hospital, School of Life Sciences and Technology, Tongji University, Shanghai, China; ^2^Department of Anesthesiology and Pain Management, Shanghai East Hospital, School of Medicine, Tongji University, Shanghai, China; ^3^Department of Biobank, Renji Hospital, School of Medicine, Shanghai JiaoTong University, Shanghai, China; ^4^Shanghai Engineering Research Center of Stem Cells Translational Medicine, Shanghai, China

**Keywords:** translation arrest, ribosome stalling, translational attenuation, erythromycin, leader peptide

## Abstract

Ribosome stalling on *ermBL* at the tenth codon (Asp) is believed to be a major mechanism of *ermB* induction by erythromycin (Ery). In this study, we demonstrated that the mechanism of *ermB* induction by Ery depends not only on *ermBL* expression but also on previously unreported *ermBL2* expression. Introducing premature termination codons in *ermBL*, we proved that translation of the N-terminal region of *ermBL* is the key component for *ermB* induced by Ery, whereas translation of the C-terminal region of *ermBL* did not affect Ery-induced *ermB*. Mutation of the tenth codon (Asp10) of *ermBL* with other amino acids showed that the degree of induction *in vivo* was not completely consistent with the data from *the in vitro* toe printing assay. Alanine-scanning mutagenesis of *ermBL* demonstrated that both N-terminal residues (R7-K11) and the latter part of *ermBL* (K20-K27) are critical for Ery induction of ermB. The frameshifting reporter plasmid showed that a new leader peptide, *ermBL2*, exists in the *ermB* regulatory region. Further, introducing premature termination mutation and alanine-scanning mutagenesis of *ermBL2* demonstrated that the N-terminus of *ermBL2* is essential for induction by Ery. Therefore, the detailed function of *ermBL2* requires further study.

## Introduction

The mechanism of bacterial resistance is most likely due to the expression of antibiotic-resistance genes. Macrolide antibiotics are used to treat infections caused by gram-positive and gram-negative bacteria (Dinos, 2017). These antibiotics hinder bacterial growth by inhibiting protein synthesis through binding to the nascent peptide exit tunnel (NPET) (Kannan et al., 2014; Patel and Hashmi, 2020). In some cases, macrolide antibiotics act not only as inhibitors of translation, but also as activators of several resistance genes (Vazquez-Laslop et al., 2008; Arenz et al., 2014; Sothiselvam et al., 2014; Almutairi et al., 2015). For example, macrolide antibiotics bind to the ribosome, promote ribosome stalling on the regulatory leader peptide *ermCL* or *ermBL*, and then activate the expression of the antibiotic resistance genes *ermC or ermB* (Vazquez-Laslop et al., 2008; Arenz et al., 2014).

The *ermB* gene encodes the ribosomal methylase that dimethylates a single adenine in 23S rRNA, which causes high-level macrolide resistance and cell survival (Chassy and Thompson, 1983; Boerlin et al., 2001; Leclercq and Courvalin, 2002). *ErmB* is often found in macrolide-resistant isolates of several gram-positive bacteria such as staphylococci, streptococci, enterococci, and clostridia, but is increasingly found in gram-negative bacteria (Schroeder and Stephens, 2016). The expression of *ermB* can be either constitutive (M19270) (Brisson-Noel et al., 1988) or inducible (M11180) (Shaw and Clewell, 1985) depending on the regulatory region located upstream of the *ermB* gene.

**FIGURE 1:**
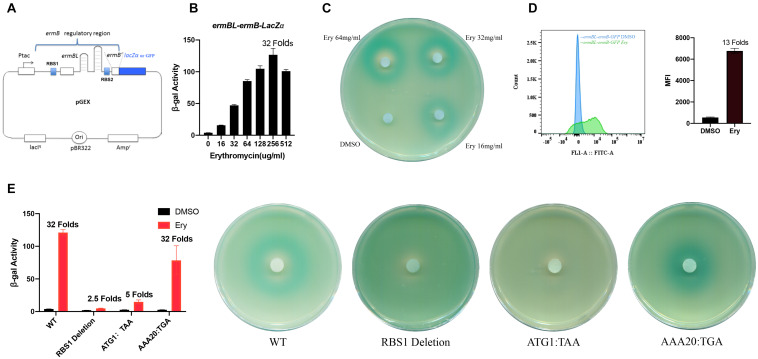
N-terminal, not C-terminal, region of *ermBL* is necessary for *ermB* induction by erythromycin. **(A)** The structure of the *ermBL-ermB*-based pGEX reporter plasmid. **(B)** β-Galactosidase activity of the *ermBL-ermB-lacZα* reporter gene during erythromycin (Ery) titration. Data are mean ± SEM from three independent experiments (unpaired two-tailed Student’s *t*-test). **(C)** Agar diffusion assays of cells transformed with the reporter plasmid containing *ermBL* grown on plates with IPTG and X-gal; each filter disc was spotted with different concentration of Ery and DMSO. **(D)** Flow cytometry of GFP intensity is shown. Data are mean ± SEM from three independent experiments (unpaired two-tailed Student’s *t*-test). **(E)** β-Galactosidase activity and agar diffusion assays of different premature terminations of *ermBL.* Plate were wetted with 1 μL of a solution of Ery (128 mg/mL).

In the *Enterococcus faecalis* strain DS16 transposon Tn917 (M11180) (Smith and Pappano, 1985), *ermB* is preceded by a 258 nt regulatory region, which contains a regulatory open reading frame *ermBL* encoding a 27 amino acid-long leader peptide. A hypothesis of translational arrest on *ermBL* as a mechanism of *ermB* induction by erythromycin (Ery) has been proved many times using the *in vitro* toe-printing assay. Specifically, the regulatory region of *ermB* includes a short leader peptide called *ermBL* with its ribosome binding site (RBS1), non-translational loop-stem structure, and several coding sequences of *ermB*, including its ribosome binding site (RBS2) (Figure 1A and Supplementary Figure 1). Without Ery, expression of *ermBL* is normal, the translation initiation site (RBS2 and AUG) of *ermB* is sequestered in the secondary structure formed by elements ➂ and ➃, and *ermB* expression is translationally attenuated (Supplementary Figure 1). In the presence of Ery, the ribosome stalls when the tenth (Asp) codon of the *ermBL* ORF enters the ribosomal P site. The stalled ribosome destabilizes helix ➀–➁ and, as a result, promotes the formation of an alternative structure of the switch region in which the translation initiation site of *ermB* is liberated and *ermB* can be translated (Min et al., 2008; Arenz et al., 2014, 2016; Dzyubak and Yap, 2016; Gupta et al., 2016; Supplementary Figure 1). However, the detailed mechanism has not yet been demonstrated *in vivo*. In addition, there are many nucleotides between elements ➁ and ➂, and their functions have not been studied.

Here, we used a well-studied *ermBL-ermB* operon (M11180) as a model to investigate the detailed induction mechanism of Ery *in vivo* (Shaw and Clewell, 1985). We constructed a pGEX*-ermBL-ermB’*-reporter plasmid. *ErmB’* is the N-terminal region of the *ermB* gene and does not have any ribosomal methylase activity, but is essential for structural transition. We found that the N-terminus, not the C-terminus, of *ermBL* was necessary for *ermB* induction by Ery. Synonymous mutation of V9-K11 of *ermBL* indicated that amino acids, rather than nucleotide sequences, are critical for induction by Ery. Mutation of D10 with other amino acids revealed that the degree of induction *in vivo* was not completely consistent with the efficiency of ribosome stalling by *in vitro* toe printing assay. Alanine-scanning mutagenesis of *ermBL* demonstrated that both N-terminal residues (R7-K11) and the latter part of *ermBL* (K20-K27) are critical for Ery induction of *ermB*. Frameshifting reporter translational fusion showed that there is a new leader peptide, *ermBL*2, in the *ermB* regulatory region. Introducing premature termination mutation and alanine-scanning mutagenesis of *ermBL2* demonstrated that the N-terminus of *ermBL2* is essential for induction by Ery. The detailed mechanism requires further research, and this study can further enhance the understanding of the mechanism of *ermB* induction by erythromycin.

## Materials and Methods

### Antibiotics, Enzymes, Chemicals, and Growth Conditions

Antibiotics (Erythromycin, Ery) were obtained from Sigma-Aldrich. Isopropyl-β-D-thiogalactopyranoside (IPTG) and 5-bromo-4-chloro-3-indolyl-D-galactopyranoside (X-Gal) were purchased from Sigma-Aldrich. Luria-Bertani (LB) broth components and agar were purchased from Sigma-Aldrich. The enzymes used for DNA cloning were obtained from Fermentas. All oligonucleotide primers were synthesized using Invitrogen DNA Technologies. *E. coli* strains were grown in LB broth at 37°C. Plasmid pGEX-4T-3(GE Healthcare) was used as the vector to generate the pGEX-*ermBL-ermB’-lacZ*α or *GFP* reporter plasmid. pGEX-*ermBL-ermB’-lacZ*α included the IPTG-inducible *tac* promoter (Ptac), followed by unique KpnI and AflII restriction sites and the transcription terminator, *lacIq* gene, ampicillin resistance (Amp^r^) marker, and pBR322 origin of replication. All the cloning procedures and most experiments with the engineered constructs were carried out with *E. coli* strain JM109 (Promega) [Genotype:*endA1, recA1, gyrA96, thi, hsdR17 (rk–, mk+), relA1, supE44*, Δ *(lac-proAB), [F’ traD36, proAB, laqIqZ*Δ *M15*].

### Construction of the pGEX Reporter Plasmid

The pGEX vector was digested with BspMI, TthIII, and a new multiple cloning site (SmaI-KpnI-XbaI-AflII-XhoI-TthIIII). The *ermBL-ermB’* cassette *(Enterococcus faecalis* strain DS16 transposon Tn917), including the tac promoter, leader ORF, and a part of the *ermB* coding sequence (*ermB’*), was directly synthesized from Invitrogen DNA Technologies. KpnI and AflII restriction sites were introduced at the ends of the amplified fragments using PCR primers. The *ermB-ermB’* cassette was cloned between the KpnI and AflII sites of the pGEX vector to produce the translational fusion plasmid pGEX-Ptac-*ermBL-ermB’*. *ErmB’* was the N-terminal region of the *ermB* gene. *ErmB’* does not have any ribosomal methylase activity, but is essential for conformational change in the proposed Ery-induced model of *ermB* (Figure 1A and Supplementary Figure 1). The reporter gene *lacZα* or *GFP* (without its start codon) was cloned into the vector following *ermB* by AflII and XhoI (Supplementary Figure 2).

### Disc Diffusion Assay of the pGEX Reporter Induction

The disc diffusion assay protocol for testing the inducibility of the pGEX reporter was carried out as described previously (Bailey et al., 2008). Briefly, the culture of cells transformed with the pGEX *lacZ*α reporter plasmid was incubated overnight in LB broth containing 100 μg/mL ampicillin. The culture was diluted 1:100 in fresh LB broth containing ampicillin (100 μg/mL) and IPTG (0.5 mM) and incubated for 1.5–2 h until the OD_600_ approached 0.2. Subsequently, 1 × 10^8^ CFU cells were added into 5 mL of 0.6% LB agar at 50°C. After brief mixing, the cell suspension was poured on top of a 1.5% LB agar plate containing 100 μg/mL ampicillin, 0.5 mM IPTG, and 160 μg/mL X-Gal. Once the soft agar had solidified, the 3 MM paper discs were placed on agar and wetted with erythromycin (128 mg/mL, 1 μL). The plates were incubated for 18–24 h at 37°C.

### β-Galactosidase Assay

*E. coli* strains carrying the pGEX-*ermBL-ermB’*- *lacZ*α plasmid were grown in LB until OD600 ≈ 0.2. Cultures were split and treated with a series of antibiotic concentrations. After treatment at 37°C for 3 h, 0.2 mL cell cultures were harvested in triplicate, and β-galactosidase activity was measured in these samples using standard protocols. Miller units were calculated by normalizing to cell density (OD_600_). At least three independent biological replicates were analyzed.

### Flow Cytometry of *GF*P Intensity

*E. coli* strains carrying the pGEX-*ermBL-ermB’*- *GFP* plasmid were grown in LB until OD600 ≈ 0.2. Cultures were split and treated with a series of antibiotic concentrations. After treatment at 37°C for 3 h, 0.2 mL cell cultures were washed with PBS and centrifuged at 500 g for 2 min. The supernatant was discarded, and the cells were resuspended in PBS. GFP levels were analyzed using a BD Aria II flow cytometer (BD Biosciences) with a 70 μm nozzle. The cell populations were detected using forward and side scatter (FSC and SSC) parameters, and fluorescence was analyzed with an emitting laser of 488 nm and a bandpass filter of 525/15 nm. Graphs were generated using FlowJo (Tree Star software), and the mean fluorescence intensity (MFI) is presented in relative fluorescence units (RFUs).

### Generation of Mutant Plasmid

pGEX-*ermBL-ermB’-lacZα* or *GFP* mutants were generated using the QuickChange site-directed mutagenesis kit (Stratagene). Mutagenic primers containing the desired mutations were amplified using Pfu Ultra DNA polymerase. The parental DNA was digested with DpnI enzyme (Fermentas). The pure mutated DNA was transformed into competent cells, and the cell suspension was poured on an LB agar plate containing 100 μg/mL ampicillin. Mutated fragments of the monoclonal bacteria were confirmed by sequencing. All the mutagenic primers sequence were showed in Supplementary Table 2.

### MIC Determinations

Minimal inhibitory concentration (MIC) determinations were performed as previously described (Xiong et al., 2005). Briefly, cells were grown overnight in LB medium containing 100 μg/mL ampicillin. On the second day, the cultures were diluted 100-fold into fresh LB medium containing ampicillin and grown for 2 h. Exponential-phase cultures were then diluted to an A600 (OD_600_ of 0.002 and placed into fresh LB medium with 3 mL/tube. After the addition of antibiotics (0, 2, 4, 8, 16, 32, 64, 128, 256, 512, 1,024, 2,048, and 4,096 μg/mL), the tubes were incubated for 15 h at 37°C without shaking and 3 h with shaking. The MIC was recorded as the lowest concentration of the drug in the tube, with no obvious turbidity.

## Results

### N-Terminus of *ermBL* Is Necessary for *ermB* Induction by Erythromycin

The expression of *ermB* is controlled by a leader ORF *ermBL* that encodes a 27-amino acid long peptide and is translationally attenuated in the absence of Ery (Min et al., 2008; Arenz et al., 2014, 2016; Dzyubak and Yap, 2016; Gupta et al., 2016). We first constructed a new pGEX reporter with the tac promoter (Ptac) following the regulatory region of the *ermBL-ermB’* operon and translational fusion to *lacZα* or *GFP* (Figure 1A and Supplementary Figure 2). The regulatory region includes *ermBL* with its ribosome binding site (RBS1), non-translational loop-stem structure, and several coding sequences of *ermB* with its ribosome binding site (RBS2) (Figure 1A and Supplementary Figure 2). *ErmB’* is the N-terminal of the *ermB* gene and has no ribosomal methylase activity, but *ermB’* is essential for structural changes in the proposed Ery-induced *ermB* model. This system allows for easy monitoring of induction by antibiotics, either by measuring β-galactosidase enzyme activity (Figures 1B,C) or *GFP* intensity (Figure 1D). *GFP* intensity is showed by histograms which *Y*-axis represents the number of cells and the *X*-axis represents the relative *GFP* fluorescence intensity of each cell. We used mean fluorescence intensity (MFI) to present in relative fluorescence units (RFUs).

Translational arrest of *ermBL* is believed to be a mechanism of *ermB* induction by Ery (Min et al., 2008; Arenz et al., 2014, 2016; Dzyubak and Yap, 2016; Gupta et al., 2016). In a previous study, *ermB* was strongly induced by the subinhibitory concentration (approximately 10–25% MIC) of Ery (Min et al., 2008; Gupta et al., 2016). We first determined the minimal inhibitory concentration (MIC) of antibiotics for *E. coli* carrying the pGEX-*ermBL-ermB’*-reporter plasmid (Supplementary Table 1). We next used our plasmid to qualitatively and quantitatively investigate the induction by Ery at a subinhibitory concentration. Erythromycin induced *ermBL-ermB’ lacZα* at a broad range of concentrations (Figures 1B,C). As expected, the antibiotic solvent DMSO did not induce *ermB* expression (Figure 1C). When we changed the reporter gene from *lacZα* to *GFP*, Ery also induced the expression of *ermBL-ermB’ GFP.* These results showed that Ery specificity induces *ermB* expression, regardless of the reporter gene.

To prove that *ermBL* expression is critical for *ermB* induction by Ery in our reporter system. We constructed some mutations impaired the expression of complete *ermBL.* The leader ORF *ermBL* translated 27 long amino acids. Therefore, we deleted the RBS1 sequence (GGAGG) or changed the first codon ATG to a stop codon (ATG1:TAA) to stop expression of *ermBL*, as expect, *ermB* induction by Ery was impaired (Figure 1E) which means *ermBL* expression is critical for *ermB* induction by Ery. When we changed the 20th codon of *ermBL* to a stop codon (AAA20:TGA), the C-terminus of *ermBL* is premature termination. *ermB* could also be induced by Ery, which means that the N-terminus of *ermBL* is necessary for *ermB* induction by Ery while the C-terminus (K20-K27) of *ermBL* is not important (Figure 1E).

### Ribosome Stalling Efficiency *in vitro* Is Not Consistent With the Degree of Induction *in vivo*

**FIGURE 2:**
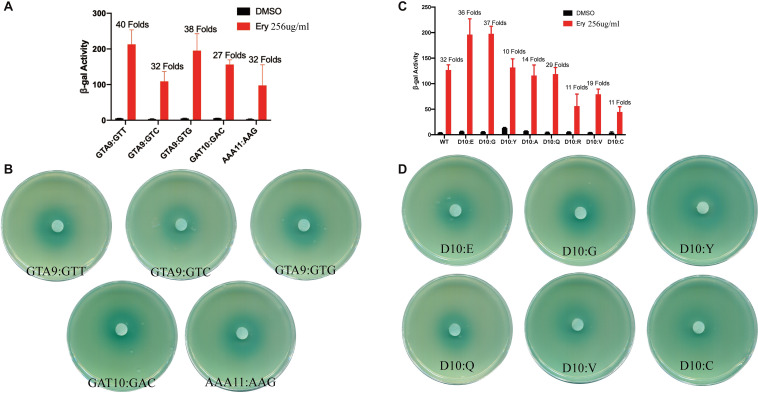
Ribosome stalling efficiency *in vitro* is not consistent with the degree of induction *in vivo.* **(A,B)** β-Galactosidase activity and agar diffusion assays of various *ermBL* synonymous mutations following Ery exposure. Data are mean ± SEM from three independent experiments (unpaired two-tailed Student’s *t*-test). **(C,D)** β-Galactosidase activity and agar diffusion assays of the degree of Ery induction *in vivo* for the Asp10 mutation of *ermBL*. Data are mean ± SEM from three independent experiments (unpaired two-tailed Student’s *t*-test).

Ribosome stalling is believed to depend on amino acid sequences but not nucleotide sequences (Min et al., 2008; Vazquez-Laslop et al., 2008; Dinos, 2017). Previous study showed that the antibiotic does not interact directly with *ermBL*, but rather redirects the path of the peptide within the tunnel (Arenz et al., 2014) which remind us ribosome stalling on *ermBL* may be amino acid-dependent. To check whether ribosome stalling on *ermBL* is amino acid-dependent, we changed the V9-K11 codon to synonymous mutations. We changed the ninth codon of *ermBL* to synonymous mutations (GTA9:GTT, GTA9:GTC, GTA9:GTG), tenth codon GAT to GAC, 11th codon AAA to AAG separately. β-galactosidase assay and disc diffusion assay showed reporter genes were induced by Ery with all synonymous mutations (Figures 2A,B), indicating that ribosome stalling on *ermBL* was amino acid-dependent.

The tenth codon Asp of *ermBL* is a key amino acid for ribosome stalling, and a previous *in vitro* study showed that ribosome stalling efficiency could be altered by mutation of the tenth codon with other amino acids (Gupta et al., 2016). Therefore, we investigated whether changes in Asp10 that affect ribosome stalling *in vitro* would result in similar changes in -sensitive gene expression *in vivo*. Gupta et al. showed that the stalling efficiency is Asp10 > Glu10 > Gly10 > ^……^ > Tyr10 > Ala10 > Gln10 > Arg10 > Val10 > Cys10 by *in vitro* toeprinting assay (Gupta et al., 2016). The stalling efficiency of most amino acids *in vitro* is consistent with sensitivity to Ery *in vivo.* The Asp10, Glu10, Gly10 mutations showed residual stalling with Ery *in vitro* also showed high induction with Ery *in vivo*, whereas Arg10, Cys10 mutations greatly diminished ribosome stalling in response to Ery *in vitro* also showed low induction with Ery *in vivo* (Figures 2C,D). β-gal activity showed that Val10 and Gln10 were also sensitive to Ery *in vivo* (Figures 2C,D), while Val10 and Gln10 prevented sufficiently strong stalling *in vitro* in previous study (Gupta et al., 2016). These results indicate that there may be other mechanisms that are independent of ribosome stalling on *ErmBL* for Ery-induced *ermB*.

### Alanine-Scanning Mutagenesis of *ermBL* Confirms Key Amino Acids for Ribosome Stalling

**FIGURE 3:**
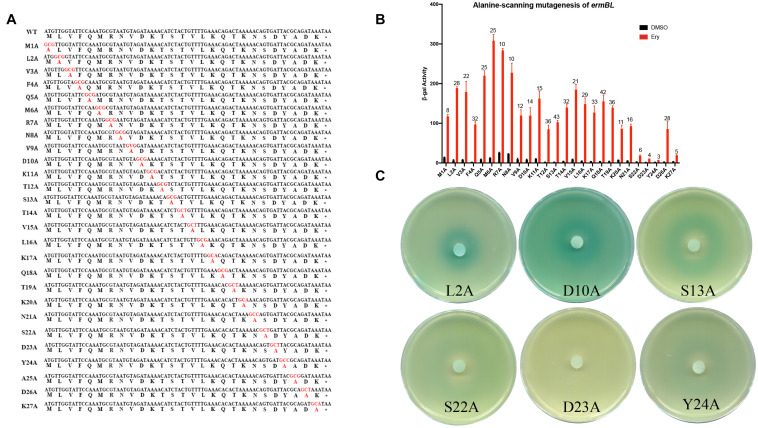
Alanine-scanning mutagenesis of *ermBL* confirms key amino acids for ribosome stalling. **(A)** Amino acid sequences of erm*BL* peptide (WT) and its alanine-scanning mutations. **(B,C)** β-Galactosidase activity and agar diffusion assays of the degree of induction by Ery *in vivo* following Ala mutation of *ermBL* amino acid sequences. Data are mean ± SEM from three independent experiments (unpaired two-tailed Student’s *t*-test).

In a previous study, the seventh to the eleventh codons of *ermBL* (R7-K11) were found to be key amino acids for translation arrest by Ery *in vitro* (Arenz et al., 2014). To determine which amino acids are necessary for *ermB* induction by Ery *in vivo*, we performed an alanine-scanning mutagenesis assay *in vivo* (Figure 3A). As expected, R7-K11 mutated to alanine impaired *ermB* induction by Ery which means R7-K11 are critical for *ermB* induction by Ery (Figures 3B,C and Supplementary Figure 7A). Unexpectively, the last codons of *ermBL* (Ser22, Asp23, Tyr24, Lys27) mutated to alanine severely impaired *ermB* induction by Ery showed these last codons of *ermBL* were also determined to be critical for *ermB* induction (Figures 3B,C and Supplementary Figure 7A). When we changed the 20th codon to a stop codon (AAA20:TGA), *ermB* could be induced by Ery, suggesting that the last codons of *ermBL* (S22-K27) are not important (Figure 1E). However, alanine-scanning mutagenesis of *ermBL* showed that the last codons of *ermBL* (S22-K27) are very important for Ery-induced *ermB* (Figures 3B,C). These contradictory results indicate that there may be another mechanism for Ery-induced *ermB*.

### A New Leader Peptide Exists in *ermB* Regulatory Region

**FIGURE 4:**
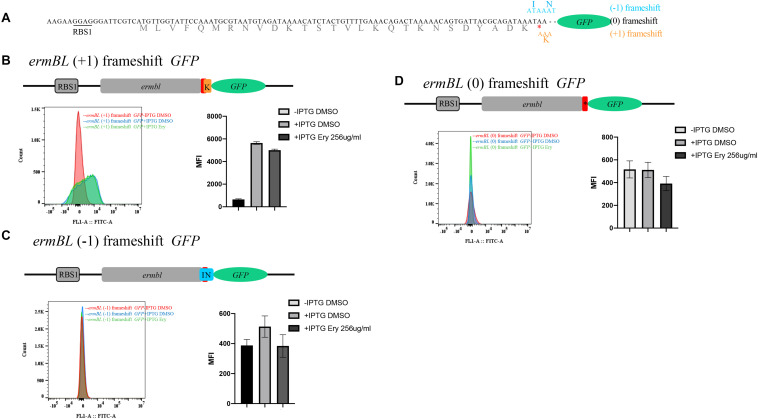
*ermBL GFP* frameshift mutation reveals new leader peptide. **(A)** The structure of the *ermBL* reporter for testing erythromycin-dependent frameshifting. **(B)** Structure of the *ermBL* (+1) frameshift reporter, *ermBL* with TAA codon following two nucleotides (AT) fused to *GFP*. *GFP* does not have its own start codon. Flow cytometry of *GFP* intensity is shown. **(C)** Structure of the *ermBL*(0) frameshift reporter, *ermBL* with a TAA codon directly fused to *GFP*. *GFP* does not have its own start codon. Flow cytometry of GFP intensity is shown. **(D)** The structure of the *ermBL*(−1) frameshift reporter, *ermBL* with TAA codon following one nucleotide **(A)** fused to *GFP*. *GFP* does not have its own start codon. Flow cytometry of GFP intensity is shown. Data are mean ± SEM from three independent experiments (unpaired two-tailed Student’s *t*-test).

Because the last codons of *ermBL* are critical for *ermB* induction, we examined other mechanisms to control the *ermB* expression by Ery. Regulation of *ermC* gene expression by ketolides is controlled by ribosomal frameshifting (Gupta et al., 2013). In this mechanism, the last codons of *ermCL* are critical for ketolide-induced *ermC* induction (Gupta et al., 2013). Therefore, we constructed an *ermBL-GFP* frameshift mutation reporter (Figure 4A and Supplementary Figure 3). In brief, the Ptac *ermBL*(0) frameshift *GFP* fusion included the tac promoter, RBS1 sequence, *ermBL* leader peptide with TAA codon, and *GFP* reporter gene (*GFP* does not have its own start codon) (Figures 4A,D). Without any (0) frameshift induced by Ery, *GFP* could not be expressed with *ermBL* expression. Ptac *ermBL*(−1) frameshift *GFP* fusion contained the same sequence as Ptac *ermBL*(0) frameshift *GFP* fusion, except with inclusion of two nucleotides (AT) behind the stop codon TAA; therefore, without any (−1) frameshifting induced by Ery, *GFP* could not be expressed with *ermBL* expression (Figures 4A,C). Further, the Ptac *ermBL*(+1) frameshift *GFP* fusion had the same sequence as Ptac *ermBL*(0) frameshift *GFP* fusion, except for one nucleotide (A) behind the stop codon TAA; therefore, without any (+1) frameshifting induced by Ery, *GFP* could not be expressed with *ermBL* expression (Figures 4A,B). All these reporter fusions were repressed by the lacI gene. Without IPTG, *ermBL* transcription was repressed.

Flow cytometry showed that (0) frameshifting, (−1) frameshifting and (+1) frameshifting mutation reporters did not induce *GFP* expression by Ery with IPTG, indicating that Ery cannot induce these types of frameshifting (Figures 4B–D). At the same time, *GFP* expression was almost at the same level with IPTG compared to that without IPTG in (0) frameshifting and (−1) frameshifting reporters, while *GFP* expression was higher with IPTG than without IPTG in the (+1) frameshifting construct (Figures 4B–D), which indicated that there was another leader peptide that (+1) frameshift with known leader peptide *ermBL*.

### Complete Sequence Information of New Leader Peptide Named *ermBL2*

**FIGURE 5:**
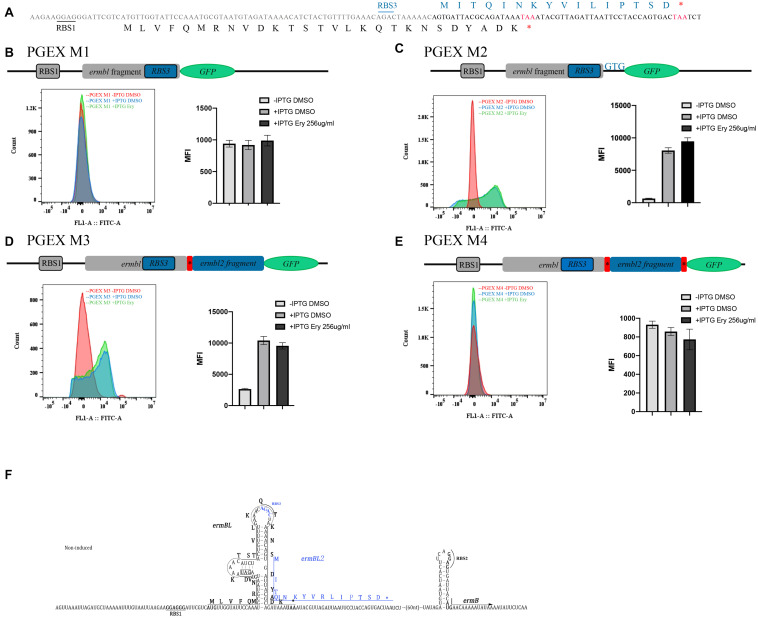
Complete sequence information of new leader peptide named *ermBL2.* **(A)** The detailed sequence of *ermBL* and predicted new leader peptide. **(B)** The structure of PGEX M1. The fusion contains the predicted RBS3, but does not have the predicted start codon. *GFP* (without its own start codon) is not expressed with *ermBL*, as *ermBL* and *GFP* have (+1) frameshifts. Flow cytometry of *GFP* intensity is shown. **(C)** The structure of PGEX M2. The fusion contains both the predicted RBS3 and predicted start codon. **(D)** The structure of PGEX M3. The fusion contains the predicted RBS3, predicted start codon, and full length of predicted *ermBL2* except the TAA codon. **(E)** The structure of PGEX M4. The fusion contains the predicted RBS3, predicted start codon, and full length of predicted *ermBL2* including the TAA codon. **(F)** Model of the *ermB* regulatory region, including two leader peptides. Data are mean ± SEM from three independent experiments (unpaired two-tailed Student’s *t*-test).

In the above study, we identified a new peptide in the *ermB* regulatory region. According to the genetic code triplet principle, start codon (ATG, TTG, or GTG), stop codon (TAA, TAG, or TGA), and (+1) frameshift with known leader peptide *ermBL*, we predicted the sequence information of a new leader peptide named *ermBL2* (Figure 5A and Supplementary Figure 4). Based on our predicted sequences, we constructed four clones to verify the complete sequence of the new leader peptide. PGEX M1 included the RBS1 sequence, a part of *ermBL*, and translational fusion to *GFP* without the start codon of predicted *ermBL2*. *ermBL* and *GFP* contained (+1)frameshift mutations; therefore, *GFP* could not be expressed with *ermBL* expression (Figure 5B). PGEX M2 included the predicted start codon (GTG) of *ermBL2* and translational fusion to *GFP* (Figure 5C). PGEX M3 did not have a stop codon of predicted *ermBL2, and ermBL2* is translational fusion *to GFP* (Figure 5D). PGEX M4 had a stop codon of *ermBL2* following translational fusion to *GFP* (Figure 5E). All the mutations were controlled by tac promoter, Without IPTG, *GFP* couldn’t be expressed. At the same time, all the fusions were (+1) frameshift with *ermBL*, therefore, *GFP* could not be expressed with *ermBL* expression. Without IPTG, mean fluorescence intensity (MFI) of all four clones was very low (Figures 5B–E). With IPTG, PGEX M2 and PGEX M4 had higher *GFP* expression than those without IPTG (Figures 5C,D), indicating that the new leader peptide named *ermBL2* is from GTG to TAA (MITQINKYVILIPTSD) (Figure 5F).

### Erythromycin Does Not Affect Expression of Leader Peptide

**FIGURE 6:**
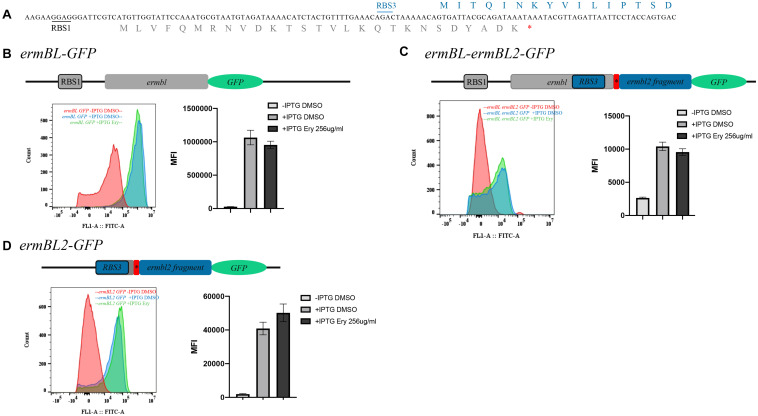
Erythromycin has no effect on leader peptide expression. **(A)** The detailed sequence of *ermBL* and the new leader peptide named *ermBL2*. **(B)** The structure of the *ermBL-GFP* translational fusion. The fusion contains the RBS1 sequence and full length of *ermBL* without the TAA stop codon. Flow cytometry of *GFP* intensity is shown. **(C)** The structure of the *ermBL-ermBL2-GFP* translational fusion. The fusion contains the full length of *ermBL and ermBL2* without the stop codon of *ermBL2.* Flow cytometry of *GFP* intensity is shown. **(D)** The structure of the *ermBL2-GFP* translational fusion. The fusion contains the RBS3 sequence and full length of *ermBL2* without the TAA stop codon. Flow cytometry of *GFP* intensity is shown. Data are mean ± SEM from three independent experiments (unpaired two-tailed Student’s *t*-test).Figure 7 | N-terminal region of *ermBL2* is necessary for *ermB* induction by erythromycin. **(A)** Amino acid sequences of erm*BL2* peptide (WT) and its alanine-scanning mutations. **(B)** β-Galactosidase activity and agar diffusion assays of the degree of Ery induction *in vivo* following Ala mutation of *ermBL2* amino acid sequences. **(C)** β-Galactosidase activity and agar diffusion assays of the degree of Ery induction *in vivo* following introduction of premature termination codons in *ermBL2*. Data are mean ± SEM from three independent experiments (unpaired two-tailed Student’s *t*-test).

Ribosome stalling on *ermBL* is a major mechanism of Ery-induced *ermB* induction. The subinhibitory concentration (25% of the minimal inhibitory concentration, MIC) of Ery induced *ermB* expression (Min et al., 2008). Macrolides selectively inhibit translation of a subset of cellular proteins rather than global inhibitors of protein synthesis and that their action most depends on the nascent protein sequence and on the antibiotic structure (Kannan et al., 2012; Almutairi et al., 2017). On the other hand, Ery does not inhibit *ermBL* expression, which is a prerequisite for *ermB* induction by Ery. We wanted to determine the effect of Ery on the expression of the leader peptide. Thus, we constructed three translational fusions (*ermBL1 GFP, ermBL1-ermBL2 GFP*, and *ermBL2 GFP*) (Figures 6A–D and Supplementary Figure 5). All three fusions were IPTG-dependent. Without IPTG, MFI of three fusions is very low (Figures 6B–D). We found that the subinhibitory concentration of Ery did not have any effect on the expression of the leader peptides as MFI of fusions is almost same with Ery compared to DMSO (Figures 6B–D). These results showed that ribosome stalling on the leader peptide may not be a stationary state, as ribosome stalling on *ermBL* does not affect *ermBL* expression.

### N-Terminus of *ermBL2* Is Necessary for *ermB* Induction by Erythromycin

**FIGURE 7:**
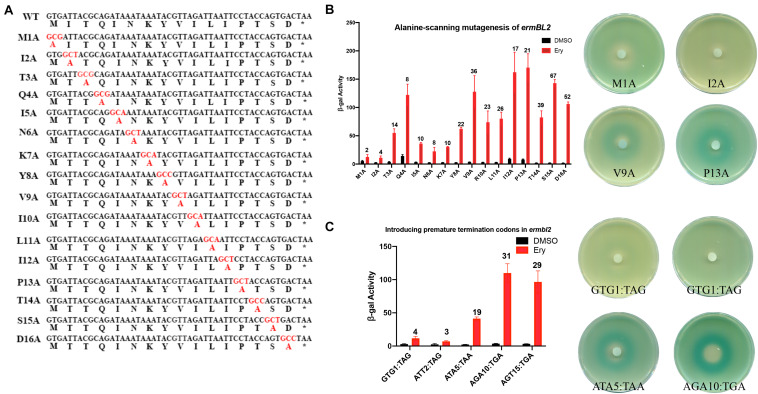
**(A)** Amino acid sequences of ermBL2 peptide (WT) and its alanine-scanning mutations. 720 **(B)** β-Galactosidase activity and agar diffusion assays of the degree of Ery induction in 721 vivo following Ala mutation of ermBL2 amino acid sequences. **(C)** β-Galactosidase 722 activity and agar diffusion assays of the degree of Ery induction in vivo following 723 introduction of premature termination codons in ermBL2. Data are mean ± SEM from 724 three independent experiments. (unpaired two-tailed Student t test).

In this study, we identified a new peptide, *ermBL2*, and wanted to determine whether its expression was critical for *ermB* induction by Ery. Alanine-scanning mutagenesis of *ermBL2* showed that the N-terminus of *ermBL2* was necessary for *ermB* induction by Ery as several amino acid of *ermBL2* mutated to alanine severely impaired *ermB* induction by Ery (Figures 7A,B and Supplementary Figure 7B). Especially, N-terminal of *ermBL2* is necessary for *ermB* induction by erythromycin (Figure 7B and Supplementary Figure 7B). Introducing premature termination codons in *ermBL2* also led to the same conclusion which N-terminal of *ermBL2* is necessary for *ermB* induction by erythromycin (Figure 7C). Taken together, these results may explain the contradictory phenomenon found in the above study: introducing a termination codon showed that the last codons of *ermBL* are not important for *ermB* induction by Ery (Figure 1E), whereas alanine-scanning mutagenesis of *ermBL* showed that the last codons of *ermBL* are very important (Figure 3B). Alanine mutagenesis of the last codon of *ermBL* not only changed the amino acids of *ermBL* but also changed the N-terminal amino acids of *ermBL*2 (Supplementary Figure 6), suggesting that the N-terminus of *ermBL* and *ermBL2* are synergetic and indispensable for Ery induction of *ermB*.

The alanine scanning experiments require at least one nucleotide changes. The Alanine-scanning mutagenesis C-terminus of *ermBL* or N-terminus of *ermBL*2 could affect the interaction between region ➁ with region ➂, which forms the stem and loop structure that releases the RBS2 and start codon of *ermB* (Supplementary Figure 1 induced). If alanine scanning experiments of *ermBL2* breaks the ➂–➃ stem structure, therefore the ➁–➂ stem structure maintains even in the presence of erythromycin which will keep low expression of their reporter genes. Under this possibility, *ermB* could not be induced by Ery just because of lost of structure change but not function of *ermBL2.* In order to exclude this possibility, we carried out a single nucleotide mutation in the start codon of *ermBL2*(GTG) to ATG, TTG, CTG, GAG, GGG, GCG, GTA, GTT, or GTC. The single base mutation has the least damage to the interaction between region ➁ with region ➂. *ErmB* could be induced by Ery with ATG, TTG or CTG mutations while other single base mutation impaired *ErmB* induction by Ery (Supplementary Figure 8B). Except GTG could be as start codon, ATG, TTG, or CTG also could be as start codon. These single nucleotide mutations showed expression of *ermBL2* is major reason for *ErmB* induction by Ery. We also changed the start codon of *ermBL2*(GTG) to alanine codon (GCT,GCC,GCA) (Supplementary Figure 8C) or stop codon (TAA,TAG,TGA) (Supplementary Figure 8D) and found *ErmB* induction by Ery is impaired which further proves the importance of *ermBL2* expression for *ErmB* induction by Ery. The fourth codon of *ermBL2*(CAG) is in the loop structure as the model of Supplementary Figure 1 induced (Supplementary Figure 8A). Theoretically, the mutation of this amino acid does not affect the interaction between region ➁ and region ➂. We changed the fourth codon of *ermBL2* (CAG) to stop codon (TGA), alanine (GCC), arginine (CGG), proline (CCG), or leucine (CTG) (Supplementary Figure 8E). We found the fourth codon mutated to stop codon or alanine impaired the *ErmB* induction by Ery while mutated to arginine, proline and leucine maintained *ErmB* induction by Ery (Supplementary Figure 8E). These data showed the expression of *ermBL2* is critical for *ErmB* induction by Ery and is amino-acid dependent.

## Discussion

Antibiotic resistance is a growing public health concern (Bader et al., 2017). The abuse of antibiotics and the lack of new antibiotics aggravate the severity of antibiotic resistance (Ventola, 2015). The mechanism of bacterial resistance is mostly due to the expression of antibiotic-resistance genes (Pontes et al., 2018). Resistance genes can be expressed in either constitutive or inducible forms. Therefore, additional studies on the expression mechanism of inducible resistance genes may address the challenge of antibiotic resistance and delay the urgent need for new antibiotics.

Macrolide antibiotics are used to treat infections caused by gram-positive and gram-negative bacteria (Patel and Hashmi, 2020). They kill bacteria by inhibiting bacterial protein synthesis and impeding the passage of newly synthesized polypeptides through the nascent peptide exit tunnel of the bacterial ribosome. Recent data have challenged this view by showing that macrolide antibiotics can differentially affect the synthesis of individual proteins in a sequence-specific manner (Kannan et al., 2014).

Ribosome stalling on *ermBL* has been proven several times *in vitro* (Arenz et al., 2014, 2016). In this study, introduction of a premature termination codon and alanine-scanning mutagenesis of *ermBL* showed a contradictory phenomenon. Specifically, introducing a termination codon suggested that the last codon of *ermBL* is not important, while alanine-scanning mutagenesis of *ermBL* highlighted the critical importance of the last codons (Figures 1E, 3B,C). To explain this phenomenon, we constructed three frameshifting fusions (Figure 4A). We did not find any frameshifting induced by Ery (Figure 4). However, we found a new peptide, *ermBL2*, expressed in the *ermB* regulatory region (Figure 4). We then introduced translational fusion constructs to determine the start and stop codons of *ermBL2* (Figure 5). Introducing premature termination mutation and alanine-scanning mutagenesis of *ermBL2* demonstrated that the N-terminus of *ermBL2* is essential for Ery induction of *ermB* (Figure 7). Further, while the C-terminus of *ermBL* and the N-terminus of *ermBL2* share the nucleotide sequence but not the amino acid sequence. Alanine-scanning mutagenesis of the C-terminal region of *ermBL* also simultaneously altered amino acids at the N-terminus of *ermBL2* (Supplementary Figure 6), which may explain the impaired *ermB* induction by Ery with mutation of the C-terminal region of *ermBL.*

Inducible Erm-type methyltransferase genes are usually preceded by a regulatory leader peptide (ORF). Currently, only a handful of macrolide resistance genes (*ermA, ermB, ermC, ermD*, and *ermS*) have been investigated in detail (Kamimiya and Weisblum, 1988; Vazquez-Laslop et al., 2008; Ramu et al., 2011; Arenz et al., 2014; Sothiselvam et al., 2014). *ErmA* and *ermD* are believed to contain two leader peptides in the regulatory region. Even ribosome stalling takes place both on *ermAL1* and *ermAL2*, yet the interplay of the two regulatory ORFs in the control of *ermA* induction is not clearly understood. Two models have interpreted the role of *ermAL1* and ermAL2: (1) ribosome stalling at *ermAL1* activates translation of *ermAL2*, whereas ribosome stalling at *ermAL2* directly leads to the translation of *ermA*. (2) Alternatively, ribosome stalling at *ermAL1* may control mRNA stability, whereas formation of the stalled complex at *ermAL2* may regulate the translation of *ermA* (Murphy, 1985; Clarebout et al., 2001; Ramu et al., 2011). *ErmDL and ermDL2* are present in the *ermD* regulatory region. Ribosome stalling at erm*DL* is critical for Ery induction, but the role of *ermDL2* has not been explored (Gryczan et al., 1984; Kwak et al., 1991; Hue and Bechhofer, 1992). Before our study, only one leader peptide was believed to exist in *the ermB* regulatory region. Our study showed that there is another leader peptide, *ermBL2*, which is also critical for *ermB* induction (Figure 5). Therefore, the classic model of the predicted conformational switch of *ermB* induced by Ery should be adjusted (Supplementary Figure 1). To date, we do not have any biochemical data (*in vitro* toe-printing assay) or bioinformatics evidence to examine whether ribosome stalling takes place on *ermBL2*. However, it is known that *ermBL1* and *ermBL2* are synergistic and indispensable for *ermB* induction by Ery.

In addition, we found that *ermBL2* expression was repressed in the *ermBL-ermBL2* sequence, as *ermBL2-GFP* expression was higher than *ermBL-ermBL2-GFP* expression (Figure 6). However, *ermBL-ermBL2-GFP* expression could not be induced by Ery, which means that ribosome stalling on *ermBL* could not induce *ermBL2* expression. As such, the mechanism of Ery-induced *ermB* and the necessity of two leader peptides warrants further study.

Macrolides affect protein synthesis in a sequence-specific manner. In a study by Kannan et al. (2014) they used the ribosome profiling technique to analyze macrolide-induced redistribution of ribosomes on cellular mRNAs and found that genes could be grouped into three major modes by Ery treatment: inhibition of translation at early stages, translation arrest at the internal codons of the gene, and translation through the entire length of the gene. To determine whether Ery affects the expression of leader peptides, we constructed three translational fusions (*ermBL1 GFP, ermBL1-ermBL2 GFP*, and *ermBL2 GFP*) (Figures 6A–D and Supplementary Figure 5). Interestingly, Ery did not have any effect on the expression of the leader peptides, even when ribosomes stalling took place on *ermBL, ermBL* expression is also not change with Ery (Figure 6). It is an interesting phenomenon and there are two possibilities about expression of leader peptide-*GFP* fusion don’t change with Ery. On the one hand, Macrolides selectively inhibit translation of a subset of cellular proteins rather than global inhibitors of protein synthesis and that their action most depends on the nascent protein sequence and on the antibiotic structure. Macrolides maybe selectively don’t inhibit translation of leader peptide. On the other hand, expression of leader peptide-*GFP* in the presence of sublethal concentrations of Ery could be the product of free-erythromycin ribosomes moving along their open reading frames. Because it is difficult to calculate the proportion of ribosomes arrested in both *ermBL* genes with Ery, we are not sure whether the expression of leader peptide with Ery is due to free-erythromycin ribosomes moving along their open reading frames or selectively don’t inhibit translation by Ery. Therefore, knowing how erythromycin work may contribute to understand its action on leader peptide. Its mechanism needs further study.

## Data Availability Statement

The raw data supporting the conclusions of this article will be made available by the authors, without undue reservation.

## Author Contributions

SW, KJ, ZH, and WH contributed to conception and design of the study. SW, XD, and YL organized the database. KJ and LL performed the statistical analysis. WH wrote the first draft of the manuscript and sections of the manuscript. All authors contributed to manuscript revision, read, and approved the submitted version.

## Conflict of Interest

The authors declare that the research was conducted in the absence of any commercial or financial relationships that could be construed as a potential conflict of interest.
