# Supplementary material for: Translational Attenuation Mechanism of ErmB Induction by Erythromycin Is Dependent on Two Leader Peptides
Source: Front Microbiol. 2021 Jun 28;12:690744. doi: 10.3389/fmicb.2021.690744 (PMC8274638; doi:10.3389/fmicb.2021.690744)
Supplement: Supplementary file 1 [file Data_Sheet_1.docx]

Supplementary Material

# Supplementary Tables

**Supplementary Table 1.** MIC determinations of *E. coli* carrying the PGEX-*ErmBL-ErmB’-lacZα* plasmid

| Antibiotics | MIC (μg/mL) | |
| --- | --- | --- |
| Erythromycin | | 1024 |

**Supplementary Table 2.** mutagenic primers sequence used in this paper.

# Supplementary Figures

**Supplementary Figure 1.** Schematic illustration for *ermBL*-dependent regulation of *ermB* translation in the presence of erythromycin

**Supplementary Figure 2.** Detailed sequence of *Ptac-ermBL-ermB- lacZα*

**Supplementary Figure 3.** Detailed sequence of *ermBL* frameshifting

**Supplementary Figure 4**. Detailed sequences of PGEX M1, PGEX M2, PGEX M3, and PGEX M4

**Supplementary Figure 5**. Detailed sequences of leader peptide translational fusions

**Supplementary Figure 6.** Alanine-scanning mutagenesis of C-terminus of *ermBL* simultaneously alters N-terminus of *ermBL2* amino acids

**Supplementary Figure 7.** Agar diffusion assays of mutated leader peptide relative to figure 3 and 7. **(A)** Agar diffusion assays of mutated *ermBL1* relative to figure 3 **(B)** Agar diffusion assays of mutated *ermBL2* relative to figure 7.

**Supplementary Figure 8.** Mutation N-terminus of *ermBL2* to other amino acids. **(A)**the model of *ermBL2* structure in induced model. **(B)** Agar diffusion assays of cells transformed with the reporter plasmid containing *ermBL* carried out a single nucleotide mutation in the start codon of *ermBL2*(GTG) to ATG, TTG, CTG, GAG, GGG, GCG, GTA, GTT or GTC with Ery(128mg/ml). **(C)** Agar diffusion assays of the start codon of *ermBL2*(GTG) mutated to alanine codon (GCT, GCC, GCA). **(D)** Agar diffusion assays of the start codon of *ermBL2*(GTG) mutated to stop codon (TAA, TAG, TGA). **(E)** Agar diffusion assays of the fourth codon of *ermBL2*(CAG) mutated to stop codon (TGA), alanine (GCC), arginine (CGG), proline (CCG) or leucine (CTG).
