# Supplementary figures and images for: Translational Attenuation Mechanism of ErmB Induction by Erythromycin Is Dependent on Two Leader Peptides
Source: Front Microbiol. 2021 Jun 28;12:690744. doi: 10.3389/fmicb.2021.690744 (PMC8274638; doi:10.3389/fmicb.2021.690744)

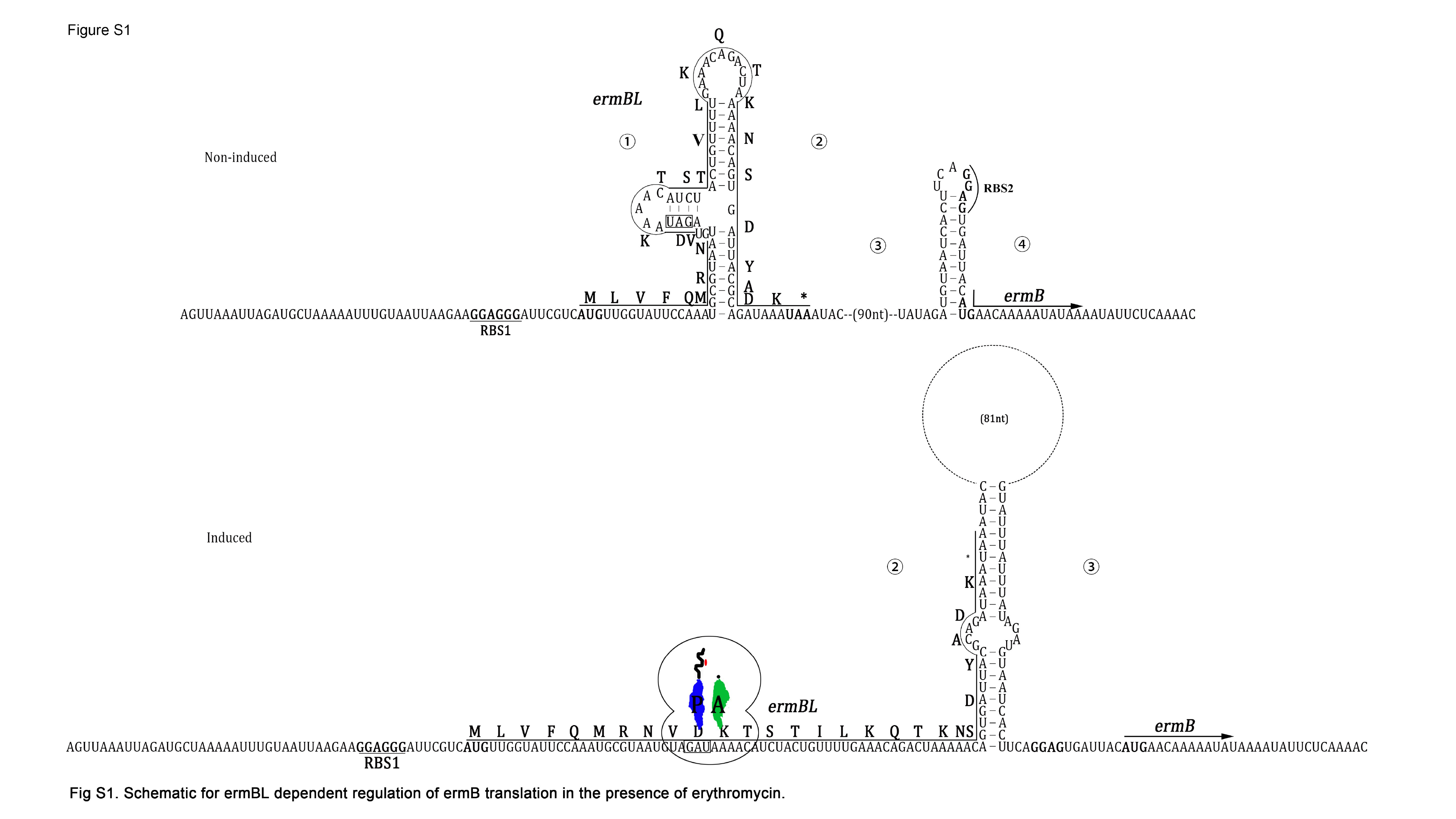

Supplement: Supplementary file 2 [file Image_1.TIF]

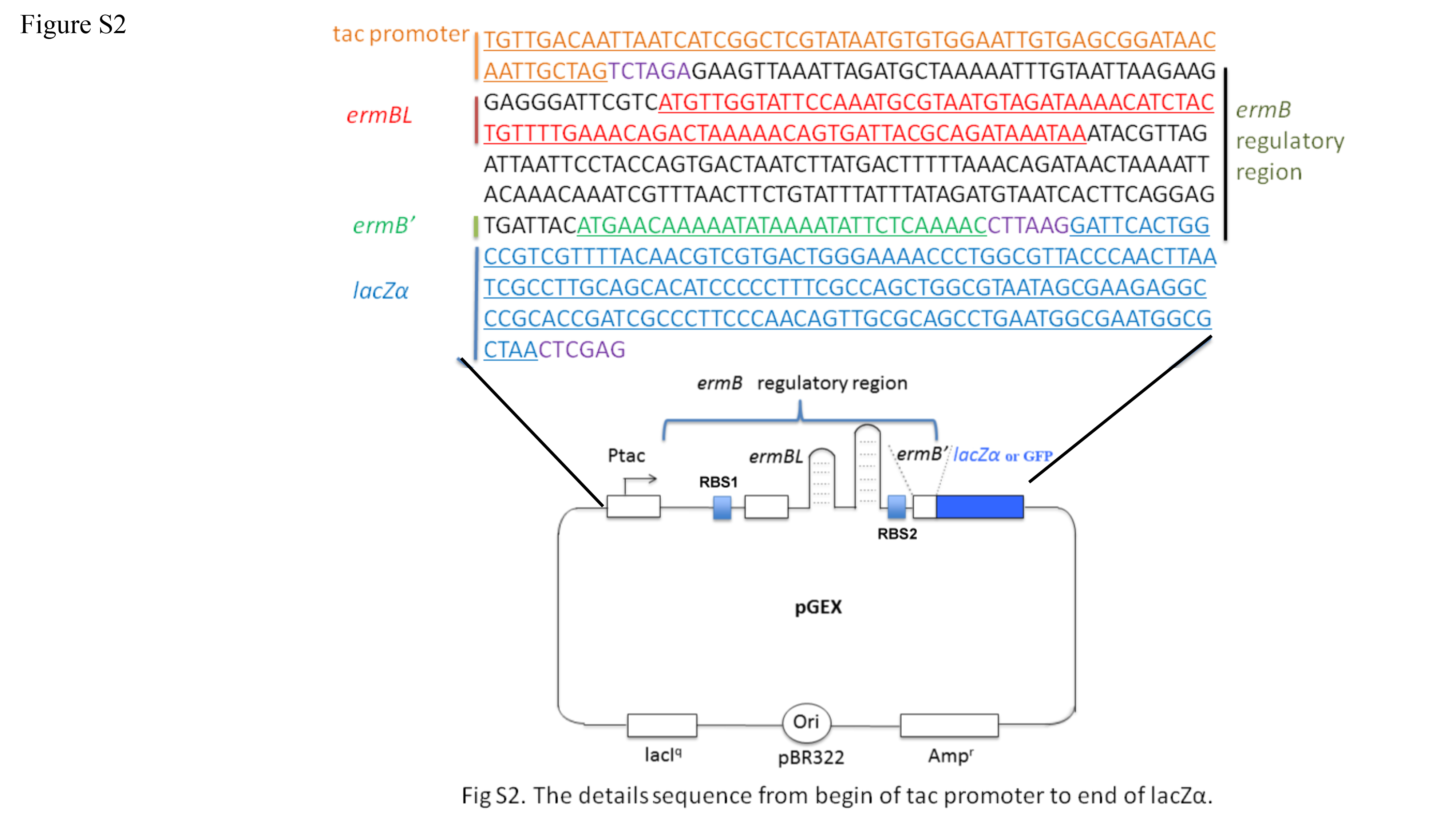

Supplement: Supplementary file 3 [file Image_2.TIF]

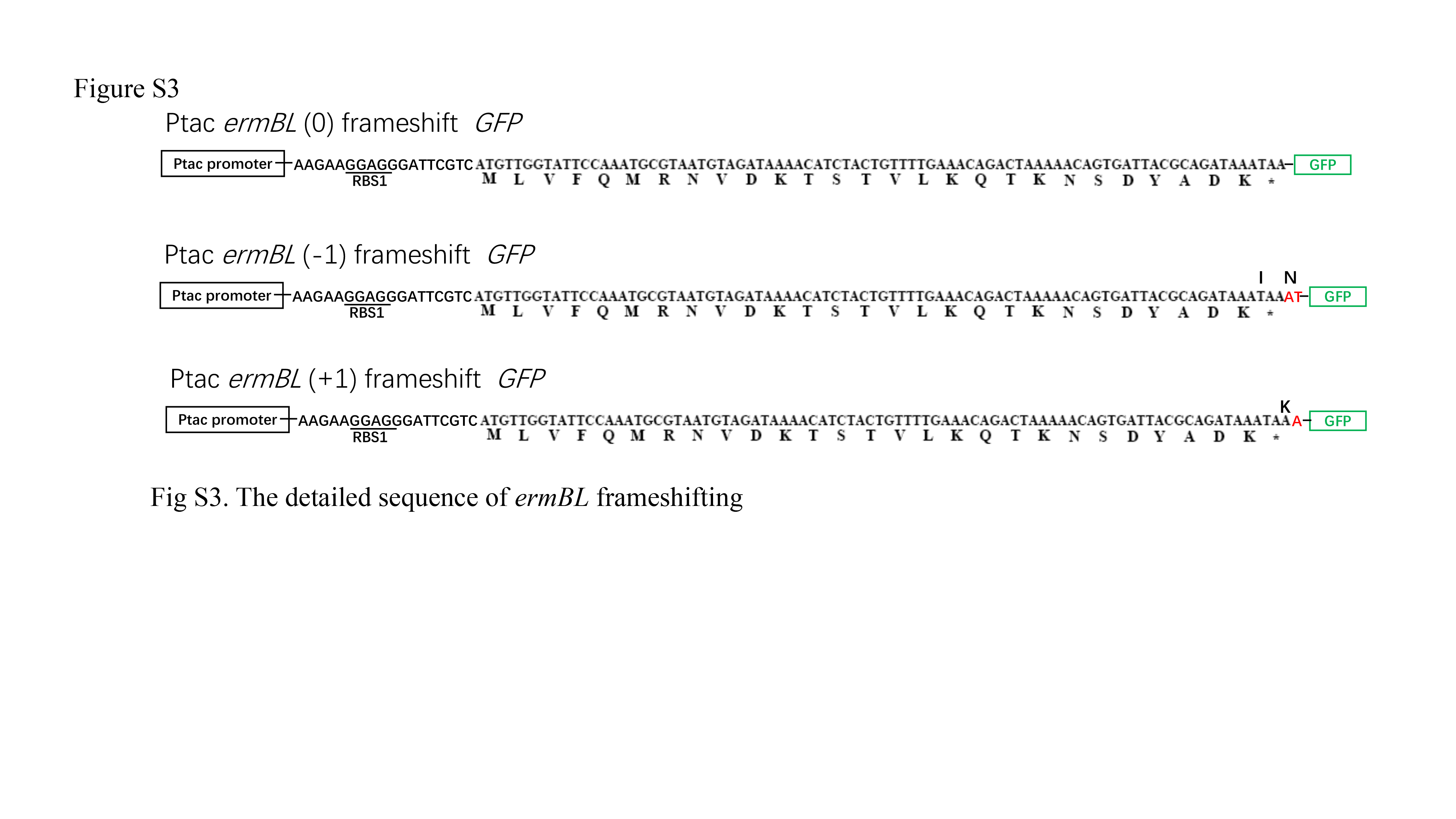

Supplement: Supplementary file 4 [file Image_3.TIF]

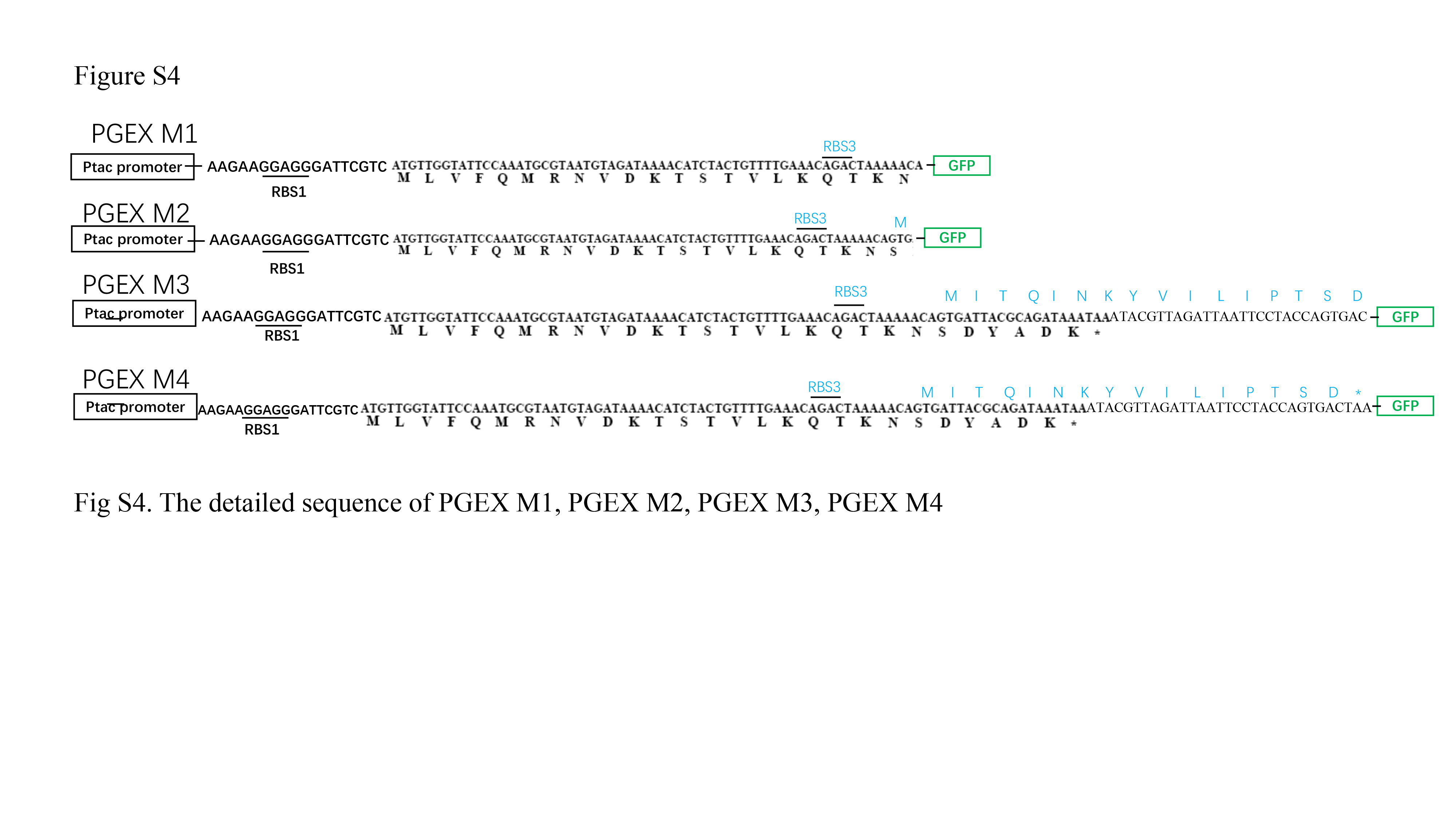

Supplement: Supplementary file 5 [file Image_4.TIF]

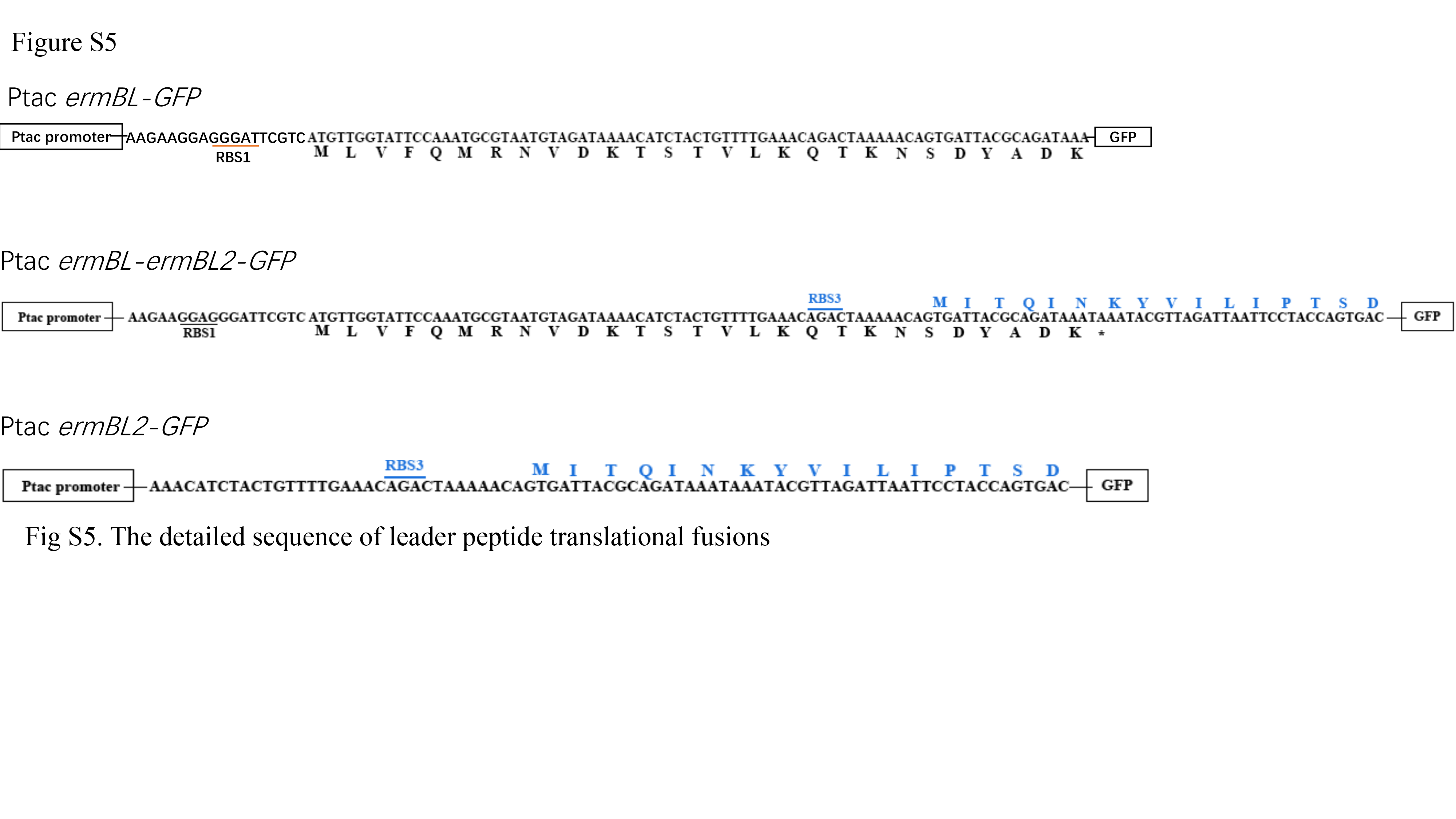

Supplement: Supplementary file 6 [file Image_5.TIF]

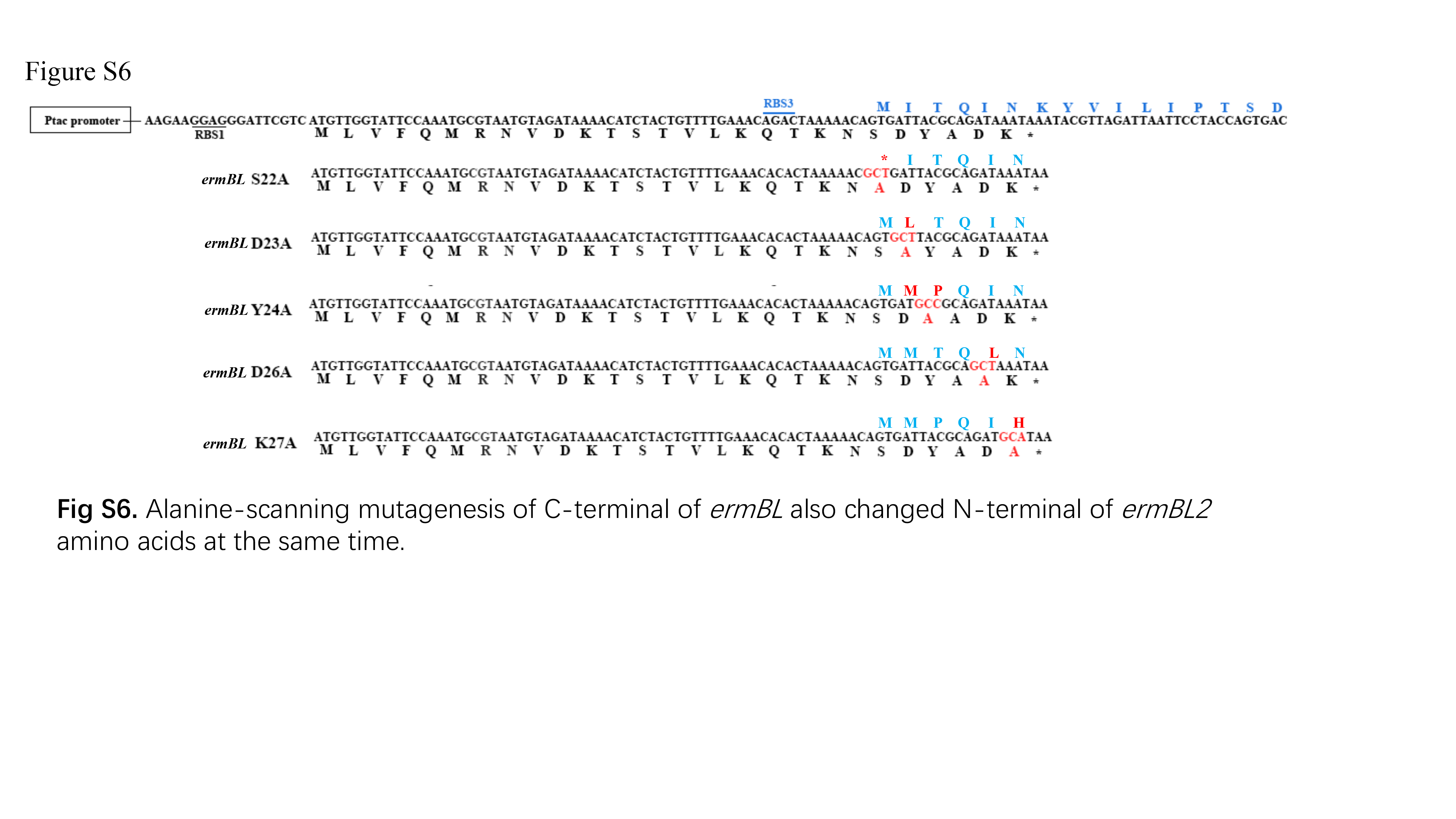

Supplement: Supplementary file 7 [file Image_6.TIF]

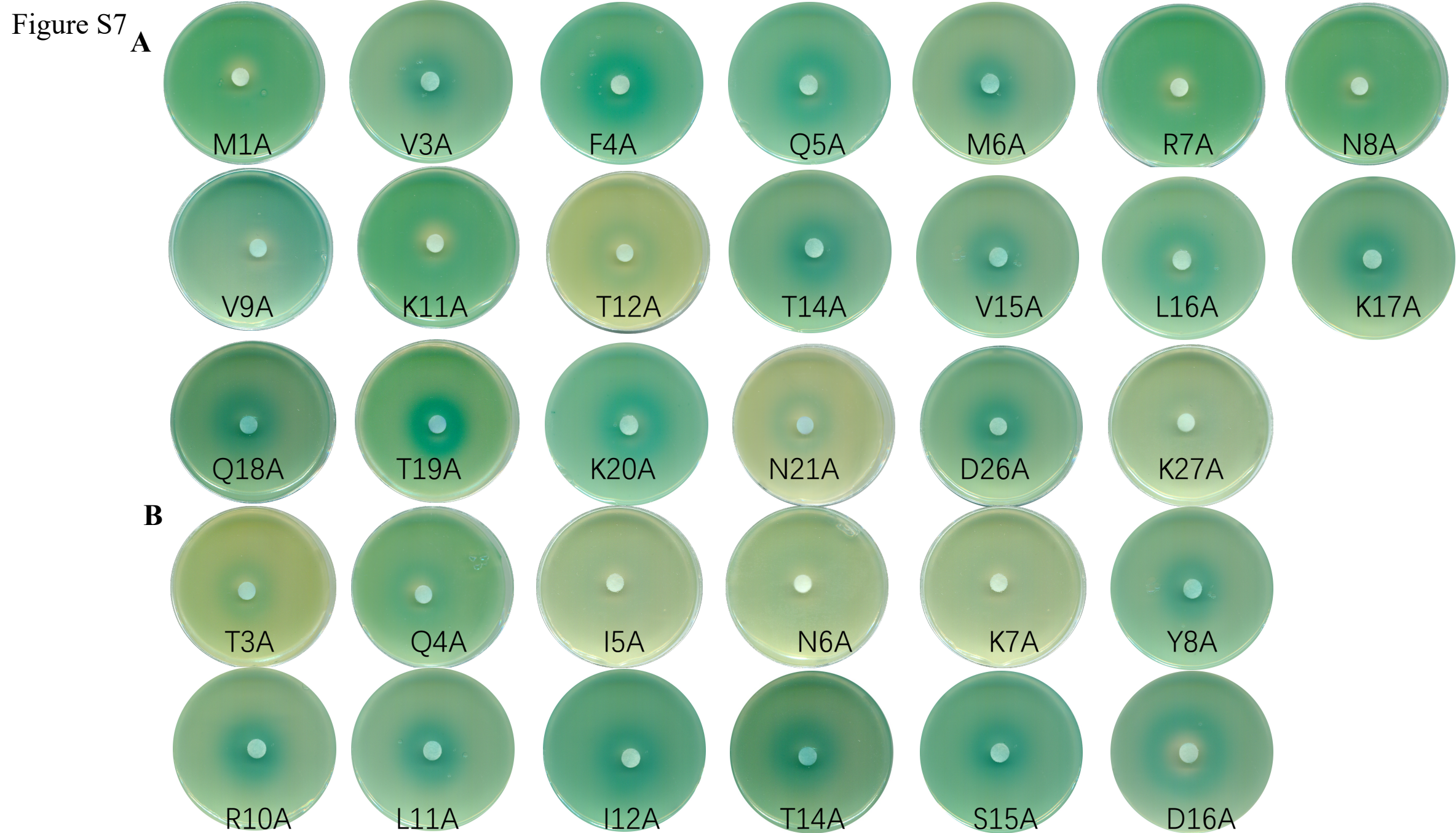

**Figure S7** Agar diffusion assays of mutated leader peptide relative to figure 3 and 7.

Supplement: Supplementary file 8 [file Image_7.PDF]

A

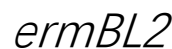

*ermB*

Supplement: Supplementary file 9 [file Image_8.PDF]
